# Wireless Acoustic Sensor Nodes for Noise Monitoring in the City of Linares (Jaén)

**DOI:** 10.3390/s20010124

**Published:** 2019-12-24

**Authors:** Jose-Angel Fernandez-Prieto, Joaquín Cañada-Bago, Manuel-Angel Gadeo-Martos

**Affiliations:** Telematic Engineering System Research Group, CEATIC Center of Advanced Studies in Information and Communication Technologies, University of Jaén, Campus Científico-Tecnológico de Linares, C.P. 23700 Linares, Spain; jcbago@ujaen.es (J.C.-B.); gadeo@ujaen.es (M.-A.G.-M.)

**Keywords:** noise monitoring, real-time noise mapping, wireless sensor networks

## Abstract

Noise pollution is a problem that affects millions of people worldwide. Over the last few years, many researchers have devoted their attention to the design of wireless acoustic sensor networks (WASNs) to monitor the real data of continuous and precise noise levels and to create noise maps in real time and space. Although WASNs are becoming a reality in smart cities, some research studies argue that very few projects have been deployed around the world, with most of them deployed as pilots for only days or weeks, with a small number of nodes. In this paper, we describe the design and implementation of a complete system for a WASN deployed in the city of Linares (Jaén), Spain, which has been running continuously for ten months. The complete system covers the network topology design, hardware and software of the sensor nodes, protocols, and a private cloud web server platform. As a result, the information provided by the system for each location where the sensor nodes are deployed is as follows: L_Aeq_ for a given period of time; noise indicators L_den_, L_day_, L_evening_, and L_night_; percentile noise levels (L_A01T_, L_A10T_, L_A50T_, L_A90T_, and L_A99T_); a temporal evolution representation of noise levels; and the predominant frequency of the noise. Some comparisons have been made between the noise indicators calculated by the sensor nodes and those from a commercial sound level meter. The results suggest that the proposed system is perfectly suitable for use as a starting point to obtain accurate maps of the noise levels in smart cities.

## 1. Introduction

Noise pollution is a problem that affects millions of people worldwide. Different studies have shown that it is currently one of the greatest environmental threats to people’s health, leading to increased risk of cardiovascular disorders, hypertension, sleep disturbance, stress, etc., and it is negatively influencing productivity and social behavior [1]. According to the World Health Organization (WHO), noise pollution is responsible for 50,000 heart attacks each year in Europe. Moreover, 1.8% of total heart attacks can be attributed to traffic noise levels greater than 60 dBA. In the particular case of Andalusia (Spain), in the studies carried out for the last Ecobarometer of Andalusia [2], citizens considered noise pollution as one of the main environmental problems in cities and towns that has caused a considerable degradation in the quality of life.

Nevertheless, until the 1990s, policies to reduce environmental noise always had a lower priority than policies regarding the pollution of water or air. In 1993, The Fifth European Commission (EC) Environmental Action Program [3] marked the beginning of attention being paid to the problem of noise pollution, and noise reduction programs began to be developed. The first step in the development of this program was in 1996, when the EC published the first policy to reduce environmental noise—“Future noise policy: European commission green paper” [4].

The Environmental Noise Directive 2002/49/EC (END) [5] required European member states to provide and publish accurate mappings of noise levels and action plans every five years throughout large agglomerations, all major roads, railways, and major airports.

Currently, noise maps can be generated with the help of noise mapping software [6,7], based on numerical simulations that take into account estimated parameters (such as traffic flow, the type of road, rail, or vehicle data), emission models of transportation and industrial noise sources, noise propagation patterns, and the urban topology.

However, the END required that noise maps be based on empirical measures. Moreover, in 2006, the EC working group “Assessment of Exposure to Noise” (WG-AEN) published a document called “Good Practice Guide for Strategic Noise Mapping and the Production of Associated Data on Noise Exposure” [8], which strongly recommended obtaining accurate and real data on noise levels.

For this task, professionals have traditionally carried out measurements using instruments for noise collection and processing, called sound level meters, placed in a mesh pattern in the area to be mapped. They measure the noise using the A-weighting equivalent continuous sound pressure level, the L_AeqT_ indicator [9].

However, this procedure has a series of disadvantages inherent to the technology used, such as the impossibility of making continuous measurements for long periods of time (weeks/months), the lack of knowledge of the situation in real time, and the inability to take preventive or corrective actions in real time. This traditional method also presents many technical difficulties for complying with some regional legislations [10].

To solve, in part, these problems and inconveniences, in recent years, different studies have proposed the use of Internet of Things (IoT)-based technologies [11].

The potential applications of IoT are numerous and diverse. In the EC documents relating to IoT [12,13,14], 65 IoT scenarios were identified and presented, grouped into 14 domains. One of these domains is the so-called smart city, defined as “a place where traditional networks and services are made more efficient with the use of digital and telecommunication technologies for the benefit of its inhabitants and business” [14]. One of the trendiest scenarios in smart cities is identified as noise urban maps—sound monitoring in bar areas and centric zones in real time.

Wireless acoustic sensor networks (WASNs) [15] play a key role in this scenario of a smart city. Over the last few years, many researchers have devoted their attention to the design of these types of networks to monitor the real data of continuous and precise noise levels, and create noise maps in real time and space. Many research works and patents have been published [16,17,18,19,20,21,22,23,24,25,26,27,28,29,30,31], but very few real projects have been developed based on WASN approaches [32,33,34,35,36,37].

In the literature [37,38], the authors present two reviews of the most relevant WASN-based approaches developed to date focused on environmental noise monitoring in smart cities. In the literature [37], WASNs have been classified according their data quality, scale, longevity, affordability, and accessibility. On the other hand, in the literature [38], another classification is presented, where the sensor nodes are divided into three main categories, according to their measurement accuracy, cost, and computational capacity.

Although WASNs are becoming a reality in smart cities, in the literature [38], the authors argue that very few projects have been deployed around the world, and they conclude that further research should be conducted to improve the performance of WASNs in real-life operation conditions. They highlight the project DYNAMAP, the objective of which is the deployment of a low-cost WASN in two different cities, Milan and Rome [36], to monitor road traffic noise.

In this work, we present the design and implementation of a complete low-cost system for a WASN deployed in the city of Linares (Jaén), Spain, which has been running continuously for ten months. The complete system covers the hardware of the sensor nodes, signal processing for noise monitoring in the sensor nodes, network topology design, protocols, and the design of a private cloud platform with an intuitive graphical user interface to show clear and comprehensible information to the general public.

As a result, a complete system has been obtained to provide the information, shown in Table 1, for each of the locations where the nodes are deployed.

In addition, along with this information, a map using the Google Maps platform Application Programming Interface (API) is also displayed, representing the L_Aeq_ in each location.

Based on the two classifications presented in the literature [37,38], the system is characterized by the following: (a) high data quality; (b) easily scalable to a large number of nodes; (c) can work continuously for long periods of time; (d) affordability due to its low-cost equipment; (e) data accessibility through a cloud web server; (f) the capacity to perform spectral analysis calculations, compute L_AeqT_, and conduct real-time signal processing; and (g) high computational capacity and low-cost equipment.

The remainder of the paper is structured as follows. Section 2 describes the complete system for the WASN deployed in the city of Linares. The experimental results are provided in Section 3. Some conclusions and future works are presented in Section 4.

## 2. The Design and Implementation of the Deployed WASN

This section describes all of the elements that make up the complete system for noise monitoring in the city of Linares (Jaén)—the network topology design, the hardware and software of the sensor nodes, protocols, and a cloud web server platform.

### 2.1. Design Considerations

**Figure 1:**
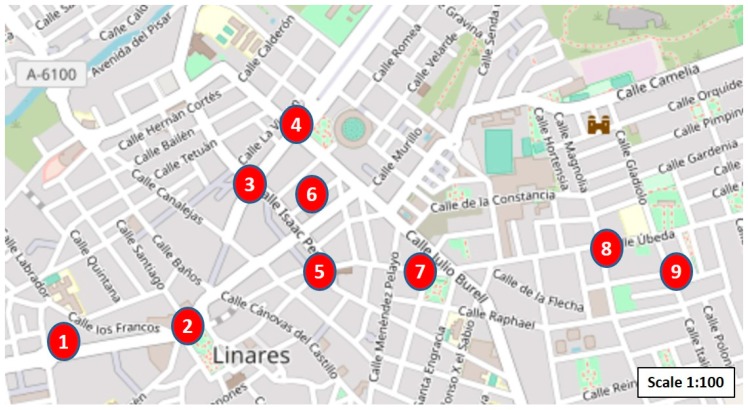
The critical locations identified for the measurement of acoustic noise in the city of Linares (Jaén).

The City Council of Linares, through the area of urban planning, established those locations of the city that were considered the most critical from the point of view of noise pollution. Specifically, eight locations were established, which mostly covered the entire downtown area. To these locations, we decided to add one more, considered as noncritical. Therefore, in total, nine low-cost nodes have been installed as measuring points. Table 2 and Figure 1 show the exact locations of these points.

The City Council of Linares specified the existence of a corporate Wi-Fi network deployed in the center area of the city, so that it was possible for the sensor nodes to transmit data. In addition, there was the possibility of using a power supply permanently at all of the measuring points.

### 2.2. Distribution of the Sensor Nodes

The topology design of a data network determines the connections between the nodes or between a node and a server. Because of the design considerations, we designed a network topology where each sensor node can send the measurements directly to a central server, which is a cloud web server in our case.

**Figure 2:**
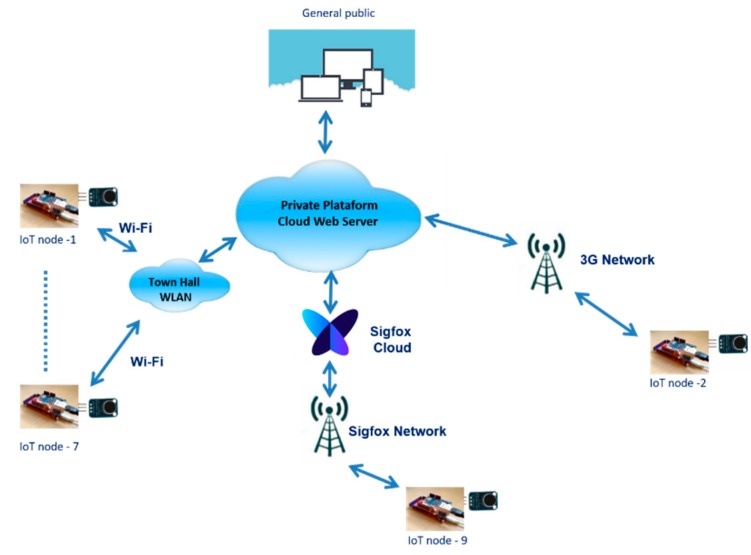
The network topology proposed for the wireless acoustic sensor network (WASN) deployed in the city.

Because of the existence of the corporate Wi-Fi network that the City Council of Linares deployed in the city, as well as the absence of power supply restrictions, we proposed using this Wi-Fi network in all of the locations where this was possible. After analyzing the coverage, it was detected that, in seven of the nine locations, it was possible to use said Wi-Fi network. However, in two locations (nodes two and nine), there was no coverage. For these two locations, we decided to use 3G and Sigfox technologies, respectively. The network topology for the proposed complete system is shown in Figure 2.

Sigfox [39] is a reliable, low-power solution based on a dedicated radio-based network to connect sensors and devices, and it needs to continuously be on and emitting small amounts of data.

### 2.3. Hardware IoT Sensor Nodes

Typical IoT devices have constrained sensor resources, an actuator capacity, and local information processing, and they are able to communicate data with servers on the Internet cloud platform.

With the design considerations indicated above, when it is possible to have a continuous power supply, the chosen device is a standard hardware model (i.e., commercial sensor node) of the Arduino platform. Specifically, it is the Arduino Due device [40], which is based on a 32-bit ARM core microcontroller, and is an open-source platform designed for the development of solutions related to sensor networks. The choice of this device is mainly due to its technical specifications, in terms of the processor and the memory, which allow for the execution of a frequency domain-based algorithm to calculate L_AeqT_ in real time. This is not possible on other devices of the Arduino platform. However, any other device with similar or better characteristics to the Arduino Due could be used, such as Raspberry Pi.

The Arduino Due has the following technical specifications: Atmel SAM3X8E ARM Cortex-M3 processor (32-bit, clock speed of 82 MHz, 96 Kb of SRAM, and 512 Kb of flash memory), 54 I/O digital ports, 12 input analog ports with a 12-bit resolution, and two output analog ports. Arduino Due hardware uses standard components, and its software is based on C/C++.

Related to the communication hardware of the sensor nodes, we used the following:

- For sensor nodes one and three through seven: Arduino Ethernet Shield [41] and an antenna MikroTiK SXT 2 [42]. We used this external antenna to ensure the existence of wireless coverage for the sensor nodes, which were connected through a UTP cable to a RJ45 female connector installed in the enclosure box. The power consumption was approximately 180 mA.
- For sensor node two: Arduino Ethernet Shield, a 3G router (model TL-MR3020 [43]), and a 3G USB modem with an outdoor antenna. In this case, the power consumption was approximately 900 mA.
- For sensor node nine: an 868-MHz Sigfox module for Arduino [44], a communication shield, and a 4.5-dBi antenna. The power consumption was approximately 125 mA.

All of the nodes were powered through passive Power over Ethernet (PoE), using 12-V 1-A power adapters and PoE injectors. The electrical plugs were at a maximum distance of 20 m, and for that distance, the voltage drop in the UTP cable was 0.9 V, so there was 11.1 V to power the Arduino, enough for its operation.

**Figure 3:**
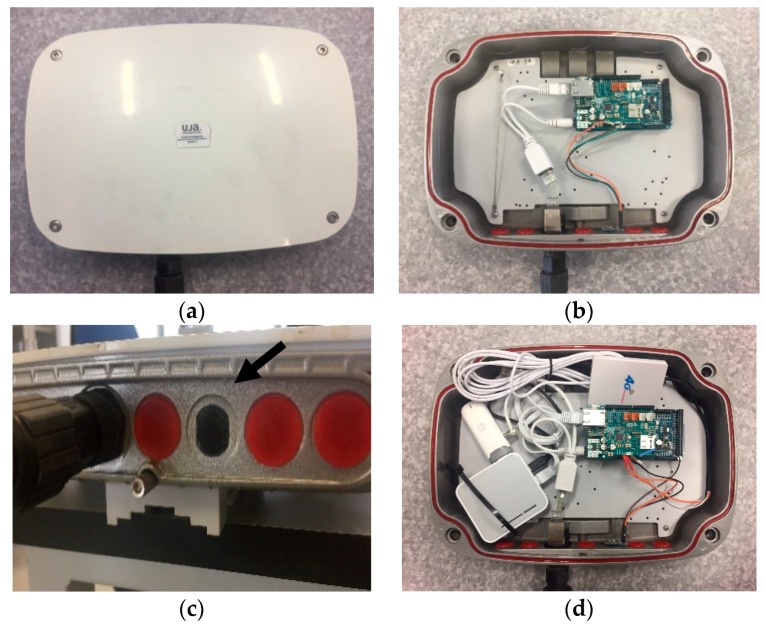
The sensor nodes: (**a**) enclosure box; (**b**) Wi-Fi sensor node; (**c**) microphone; (**d**) 3G sensor node.

The microphone used is based on a commercial design [45]. Each sensor node is equipped with an electret microphone, 20–20 kHz (Figure 3). It is much more than just a microphone, because it is integrated with an operational Maxim MAX4466 specifically designed for acoustic solutions (it amplifies and filters the noise). The gain is adjustable via an integrated potentiometer. Moreover, the microphones have a miniature foam windshield ball [46]. For the outdoor enclosure for the nodes, we used IP66-rated outdoor aluminum enclosures for wireless platforms, such as StationBox ALU RF elements [47]. Figure 3 shows some sensor nodes.

To the best of our knowledge, this system has one of the lowest economic costs per node, and this aspect is very important when implementing WASNs with a large number of nodes. Table 3 shows the costs of each node (tax not included). Costs can be more reduced by using compatible materials.

### 2.4. Software Implemented in the Sensor Nodes for Noise Monitoring

For the measurement of acoustic noise and to integrate the calculated L_AeqT_ into the sensor nodes, it is necessary to design and implement an algorithm that runs on these nodes. In the previous work [48], we presented a frequency domain-based algorithm to calculate L_AeqT_ in real time adapted to resource-constrained devices, such as wireless acoustic sensor nodes.

**Figure 4:**
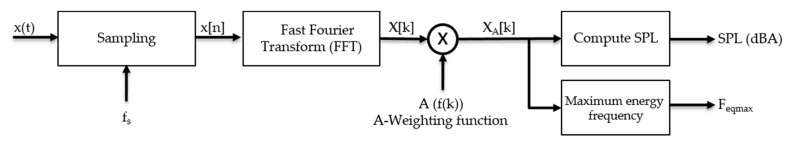
The algorithm’s functional blocks. SPL—sound pressure level.

In this work, we improved the algorithm by introducing a new module to determine the frequencies with a higher energy and their degree of importance with respect to background noise or less significant frequencies. In this manner, we obtained the information from the predominant frequency of the noise. The optimized architecture used by the algorithm consists of four functional blocks, as shown in Figure 4.

The sampling block is responsible for sampling the acoustic signal x(t). The IEC 60651 Type-2 SLM acoustic standard [49], superseded by IEC 61672 [50], established the measurement of environmental noise between 0–8 kHz, and the SLMs specified in the literature [50] are intended to measure sounds generally in the range of human hearing. As is well known, in urban areas, the acoustic signal energy is concentrated in a low-frequency region (<10 kHz). Based on this, we configured the Arduino Due with a frequency-sampling rate, *f_s_*, of 33 kHz, by a software function with a resolution of 12 bits. Thus, it is not possible to measure frequencies higher than 16.5 kHz. Related to the time-constant of the integration or time capture, there are two time-weightings that have been internationally standardized, namely: (a) slow response (S) of one second; and (b) fast response (F) of 125 ms.

The second block receives the audio samples from the first block. The algorithm is based on a frequency analysis, which uses the discrete Fourier transform (DFT) to determine the frequency spectrum of a segment of audio samples. Let us denote *X*[*k*] as the DFT of a windowed signal *x*[*n*], at the digital frequencies 2π*k*/*N* radians, where *N* denotes the number of samples and *k* = 0, …, *N* − 1. To determine the samples of the DFT, we use the following equation:

(1) $$f\left(k\right)=\frac{f_{s}\cdot k}{N}.$$

The difference between two consecutive samples is given by the following expression:

(2) $$\Delta f=\frac{f_{s}}{N}.$$

Regarding the CPU and memory requirements, this block is the most demanding. For a computationally efficient implementation, we used the fast Fourier transform (FFT) to evaluate the DFT. Some analyzers have been designed to determine the optimal FFT length and thus achieve efficient implementation. Additionally, an exhaustive analysis has been performed using software functions to the reduce memory requirements and the execution time. The FFT length depends on the frequency-sampling rate chosen and the time capture ($T_{w}$), as follows:

(3) $$N=f_{s}\cdot T_{w}$$

Table 4 shows the FFT length for the two time-weightings that have been standardized and for the frequency-sampling rate of 33 kHz.

Taking into account that the FFT is more runtime efficient if a base-two sample length is selected, to reduce the execution time, we propose a length of 4096 samples (instead of 4125) for the fast response and 32,768 samples (instead of 33,000) for the slow response. This means a slight decrease in the time window at 124.1 ms for the fast response, and at 0.993 s for the slow response. Our proposal is to perform an FFT calculation for the fast response only, which uses a time window of 124.1 ms. This approximation of the time window produces an error of 0.7% (29 samples less than using a time window of 125 ms), which could be considered as negligible. Larger FFT sizes provide a higher spectral resolution, but take more resources to compute. A smaller window size means a shorter runtime, and, therefore less resource consumption.

Once the frequency components of the acoustic signal are available, an A-weighted filtering is implemented. This filtering stage allows for weighting of the different frequency components of the acoustic signal, consistent with a typical human ear response. The mathematical function used to obtain the value of the attenuation depends on the frequency, and is given by the normative IEC 61672 [50], as follows:

(4) $$\begin{array}{c} \left(f\right)=10\log_{10}\left[\frac{1.562339^{2}\cdot f^{4}}{\left(f^{2}+107.65265^{2}\right)\left(f^{2}+737.86223^{2}\right)}\right]+\\ \;+10\;log_{10}\left[\frac{2.242881\cdot 10^{16}\cdot f^{4}}{\left(f^{2}+20.598997^{2}\right)\left(f^{2}+12194.22^{2}\right)}\right] \end{array}$$

In the above equation, *f* is the frequency and *A*(*f*) is the associated attenuation. However, some resource-constrained devices have an anomalous behavior for math operations with large numbers. Therefore, we propose the use of an equivalent expression to reduce the complexity of the mathematical operations [51].

(5) $$A\left(f\right)=2+20\;log_{10}\left(R_{A}\left(f\right)\right)$$

$$R_{A}\left(f\right)=\frac{12200^{2}\cdot f^{4}}{\left(f^{2}+20.6^{2}\right)\sqrt{\left(f^{2}+107.7^{2}\right)\left(f^{2}+737.9^{2}\right)}\left(f^{2}+12200^{2}\right)}$$

Using Equations (1) and (2), we can determine the frequency for each sample of the FFT. After applying the filter, the obtained signal is as follows:

(6) $$\;X_{A}\left[k\right]=A\left(\Delta f\cdot k\right)\cdot X\left[k\right]$$

Finally, the last block computes the total energy of the weighted frequency components to obtain the sound pressure levels (SPLs) in dBA. Using the Parseval’s relation [52], the total energy of the waveform can be summed across all of its frequency components, as follows:

(7) $$\epsilon_{x}=\frac{1}{N}\overset{N-1}{\underset{k=0}{\sum }}{\left|X\left[k\right]\right|}^{2}$$

Regarding the properties of the FFT, the samples have symmetry because they are complex conjugates, as follows:

(8) $$X\left[\frac{N}{2}+k\right]=X^{*}\left[\frac{N}{2}-k\right]\;1\leq k\leq \left(\frac{N}{2}\right)-1$$

The input samples in the time domain are real values. In the frequency domain, they are symmetrical from the sample *N*/2 (*N* is even). Therefore, we can calculate the energy of the signal using only the first *N*/2 + 1 samples (filtered spectrum with A-weighting filter). The expression is the following:

(9) $$\epsilon_{x}\approx \frac{2}{N}\overset{\left(\frac{N}{2}\right)-1}{\underset{k=1}{\sum }}{\left|X_{A}\left[k\right]\right|}^{2}+{\left|X_{A}\left[0\right]\right|}^{2}+{\left|X_{A}\left[N/2\right]\right|}^{2}$$

Equation (9) is used to determine the total energy of the signal in the time capture. Taking *T_w_* seconds, the instantaneous average power of the signal is given by the following:

(10) $$P_{x}\approx \frac{\epsilon_{x}}{\;T_{w}}$$

To obtain the *SPL* in dBA, we applied the following expression:

(11) $$SPL\;\left(dBA\right)=10\cdot log_{10}\left(P_{x}\right)+C$$

where *C* is the calibration constant, which will be calculated in the calibration process.

#### Calibration and Test Results

**Figure 5:**
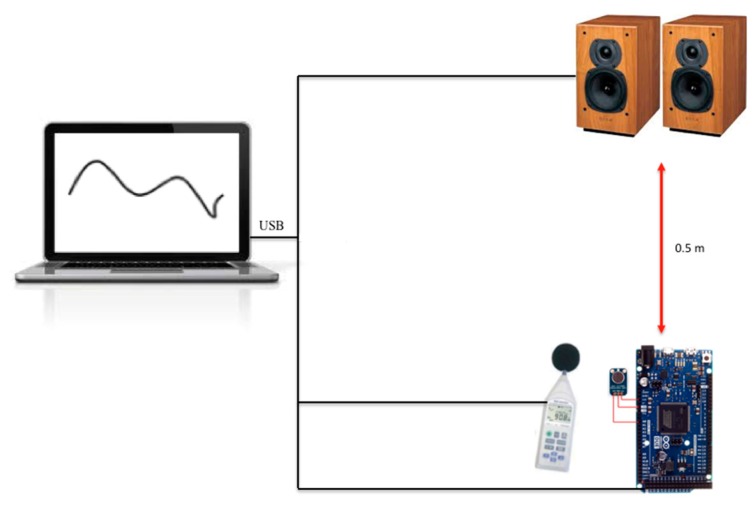
The scenario for indoor measurement tests.

Before deploying the sensor nodes in the urban area, some tests were carried out in the lab and in a street to verify the quality of the noise measurements. Figure 5 presents the lab scenario where we used an Arduino Due with the microphone, a commercial Sound Level Meter PCE-353 (SLM) [53], and one laptop with speakers. The SLM and the sensor node were connected to the laptop using a USB connection. The sensor node and the SLM were deployed closely; the distance from the speakers to the devices was 0.5 m.

**Figure 6:**
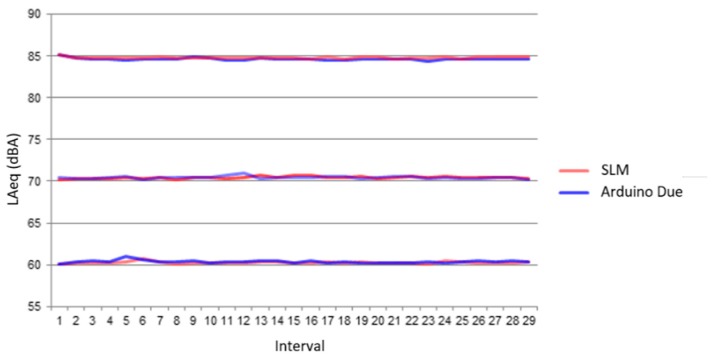
The white noise lab tests.

First, the commercial SLM was calibrated using the Class-2 Sound Level Meter Calibrator PCE-SC 42, at one kHz and for an SPL of 94 dB. Later, to calibrate the sensor node, an acoustic signal of a 1-kHz tone was created using math software. The volume of the speakers was raised until the SLM measured 94 dB, and the microphone gain was adjusted to give that measurement. Once both devices were calibrated, an acoustic signal composed of white noise (30 Hz–20 kHz) was generated with three noise levels of acoustic intensity: 60, 70, and 85 dBA. For each level, the L_Aeq_ indicator was calculated after repeating the experiment 30 times. The duration of the acoustic signal was five seconds for each test. Figure 6 shows the results for this experiment.

Table 5 shows the average value of the 30 measurements of the L_Aeq_ indicator for each level of acoustic intensity, and the absolute error (Diff) between the Arduino Due and the commercial SLM measurements.

**Figure 7:**
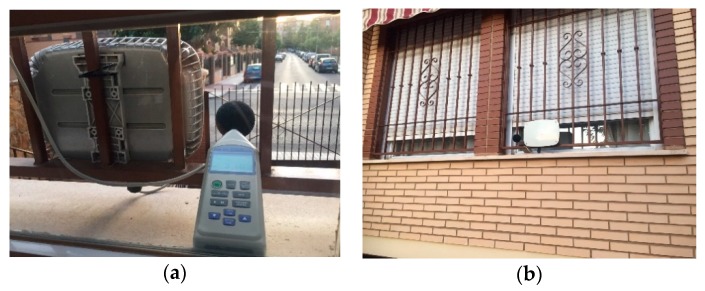
The SLM and Arduino Due deployed in a street for the tests: (**a**) back view; (**b**) front view.

As can be observed, the differences between the Arduino Due and the SLM were lower than 0.2 dBA. For another test, we deployed the SLM and Arduino Due devices in an urban street. The distance between the devices and street was approximately eight meters. The measurements were made during one hour in daytime. Both devices calculated the SPLs each second, for four intervals of 15 min each. Table 6 shows the L_Aeq_ and the absolute error (Diff) between the SLM and Arduino Due measurements. Figure 7 shows the location where the sensor and the SLM were located.

In this case, the differences between the Arduino Due and the SLM were lower than 0.9 dBA, which represents a very good agreement.

Finally, we deployed the SLM and Arduino Due devices in the same urban street, but this time for a full day. The results are shown in Table 7.

The differences between the Arduino Due and the SLM were approximately 1 dBA in the L_Aeq_ measured. The maximum difference was in the period L_evening_, being 2.41 dBA. This is because the dynamic range of the sensor is 44–105 dBA, and therefore, it cannot measure noise levels below 44 dBA. Alternately, the L_Amax_ measured with the SLM was 88.1 dBA, while that with the Arduino Due was 90.5 dBA. The results of the previous tests indicate that the software designed for the Arduino Due has a good performance when we compare the differences between the acoustic measurements calculated by the Arduino and those of the commercial SLM.

### 2.5. Protocols and Platform Cloud Web Server

Many protocols have been specifically designed for communication between IoT devices, namely: Message Queue Telemetry Transport (MQTT) [54], Constrained Application Protocol (CoAP) [55], Advanced Message Queuing Protocol (AMQP) [56], Data Distribution Service (DDS) [57], etc. However, the commonly used protocol for the Internet, Hyper Text Transfer Protocol (HTTP), is used in most cases for IoT devices when they need to publish a considerable amount of data.

In fact, most of the commercial platforms that currently exist in the cloud and intend to provide services to IoT devices allow for communication from these devices through HTTP. Some examples are the Amazon AWS IoT Core platform, Microsoft Azure, Google Cloud IoT Core, and Thingworx. Each platform offers developers a series of application programming interfaces (APIs) and software development kits (SDKs) that make it possible to establish communication between the IoT devices and the cloud platform.

The use of the services of these platforms has advantages and disadvantages. Among the drawbacks of Azure, in addition to the cost involved in its use, are that the implementation of real-time data visualization systems is sometimes complex, and there is incompatibility with the Safari web browser. Google IoT Core Cloud does not support the MQTT protocol. In the AWS IoT Core, the use of services and functions is complex in some cases, and it is the most expensive option for many services.

Therefore, in our case, we decided to design and implement our own platform cloud web server using the infrastructure of the University of Jaén. The cloud web server is based on a model–view–controller (MVC) software architecture, which separates the application data, the user interface, and the control logic into three distinct components. For frontend technologies, we used HTML5, CSS3, Bootstrap, and JavaScript, and for the backend technology, we used PHP. For the database, a MySQL relational database was designed. In addition, for the representation of the L_Aeq_ values of the locations on a map with different colors, the Google Maps platform API was used.

In all of the sensor nodes, in addition to the acoustic noise monitoring software, the communication software was implemented to send data to the Platform Cloud Web Server. To send the data, we decided to use the HTTP protocol. Therefore, a web client was implemented on each sensor, except for on sensor node nine. Node nine was programmed using the Sigfox API for sending data, and a callback was configured in the Sigfox backend, so that an HTTP request with the GET method was made to our cloud web server.

**Figure 8:**
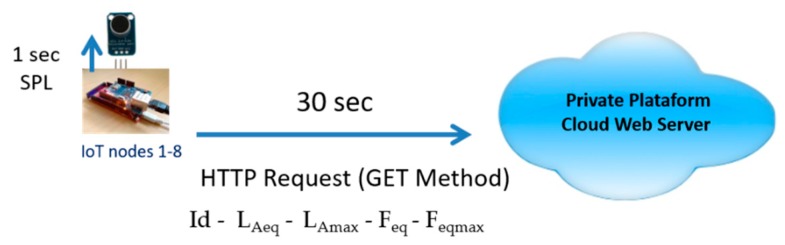
The Hyper Text Transfer Protocol (HTTP) request—GET method.

**Figure 9:**
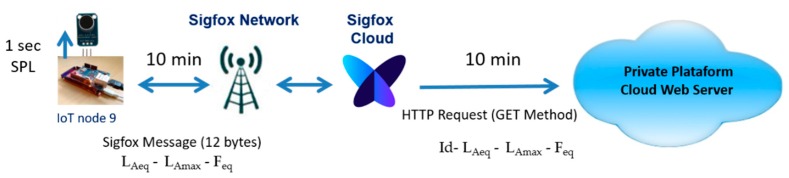
The Sigfox message. HTTP request—GET method.

As shown in Figure 8 and Figure 9, all of the sensor nodes calculate the SPL every second. Every 30 s, sensor nodes one–eight send the following data to the Platform Cloud Web Server:

1. Id sensor node
2. L_Aeq_ calculated for these 30 s
3. L_Amax_ in the period of 30 s
4. Average predominant frequency of the noise during 30 s (F_eq_)
5. Higher predominant frequency of the noise during 30 s (F_eqmax_)

In the case of sensor node nine, Sigfox messages can carry a payload (user data) of 12 bytes, and 140 messages are permitted per day, at most (although we have verified that this limit can actually be exceeded a bit). Therefore, as shown in Figure 9, sensor node nine sends a message every 10 min with the following parameters:

1. L_Aeq_ (four bytes) calculated for these 10 min
2. L_Amax_ (four bytes) in the period of 10 min
3. Average predominant frequency (four bytes) of the noise during 10 min (F_eq_)

## 3. Results

As a main result, we can say that an experimental wireless acoustic sensor network for real-time noise monitoring has been installed in the city of Linares (Jaén), Spain, and it has been running continuously for 10 months.

### 3.1. Sensor Nodes Deployed around the City

**Figure 10:**
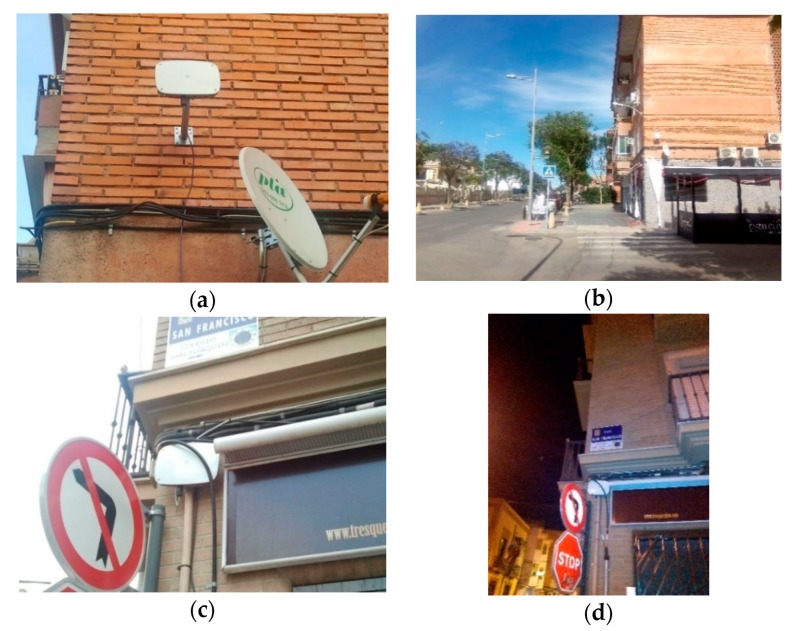

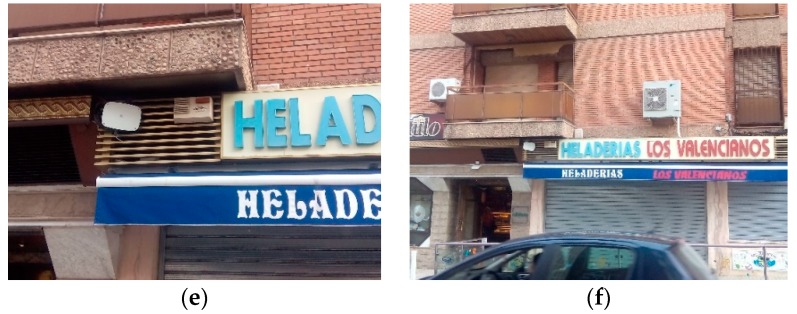
The sensor nodes deployed in the city. (**a**,**b**) Id 8: Ubeda Street; (**c**,**d**) Id 5: San Francisco Square; (**e**,**f**) Id 1: Andalucia Avenue.

Figure 10 shows some locations where the sensor nodes were installed. From the data sent to the cloud web server by the sensor nodes, a huge amount of information has been obtained, with which it would be possible to characterize the city in terms of its activity. In this section, we will show some of the results obtained, but it is impossible to show all of the cases and events that have occurred in all of the locations.

### 3.2. The Information Offered by the Cloud Web Server

**Figure 11:**
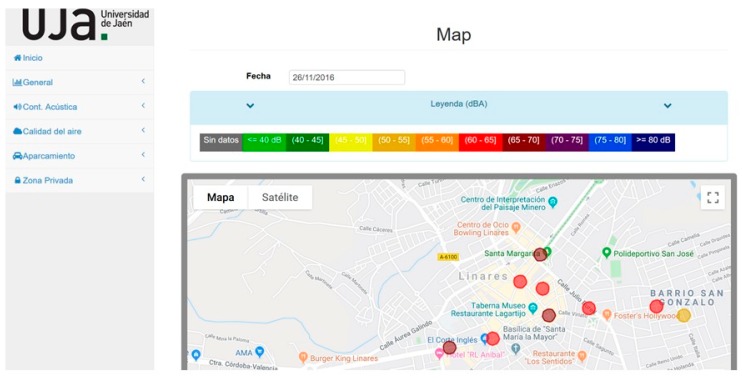
The map with the noise in the sensor locations on 26 November 2016.

The cloud web server offers the possibility of selecting a date to display a map using Google Maps, where L_Aeq_ is represented by a color in each location measured during the 24 h of the previous day. Figure 11 shows an example of city noise for the day of 26 November 2016.

**Figure 12:**
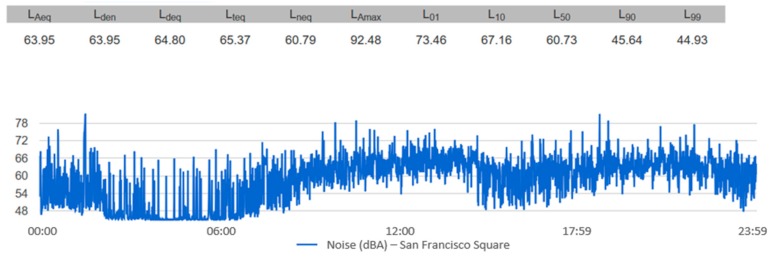
The temporal evolution of the noise from 00:00 to 23:59 on 30 June 2017 in San Francisco Square.

**Figure 13:**
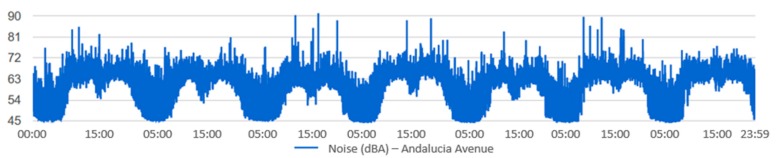
The temporal evolution of the noise in Andalucia Avenue location for a full week in the month of October.

For each one of the locations, the temporal evolution of the noise during the selected time interval can be visualized, as well as the noise indicators. Figure 12 and Figure 13 show an example of how this information is displayed. Figure 12 shows the acoustic noise measured by the sensor of San Francisco Square from 00:00 to 23:59 on 30 June 2017. Figure 13 shows the temporal evolution of acoustic noise for a full week at the Andalucia Avenue location. It can be seen that the acoustic noise has a similar pattern every day.

**Figure 14:**
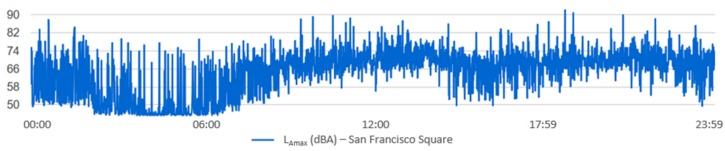
The L_Amax_ measured at each 30-s interval from 00:00 to 23:59 on 30 June 2017 in San Francisco Square.

It is possible to set the query for a certain period of time. For example, if L_Amax_ is 92.48 dBA, a query can be made to visualize the L_Amax_ that occurred in each one of the 30-s intervals, as shown in Figure 14.

**Figure 15:**
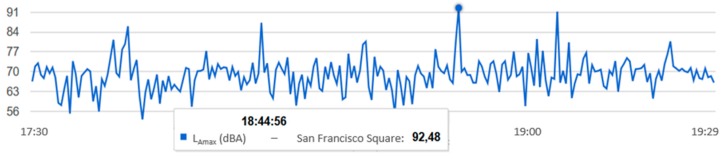
The L_Amax_ measured at each 30-s interval from 17:30 to 19:30 on 30 June 2017 in San Francisco Square.

It can be observed that the L_Amax_ of that day was produced in the strip from 17:30 to 19:30, so if we only consult that period, we can know exactly when the L_Amax_ occurred, which was at 18:44, as seen in Figure 15.

**Figure 16:**
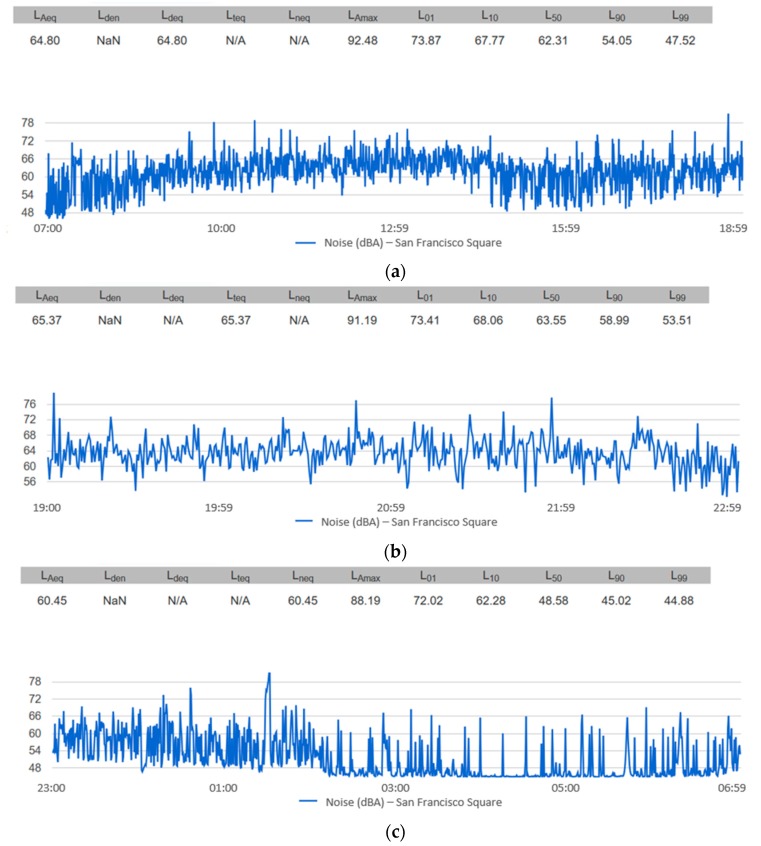
The temporal evolution of the noise on 30 June 2017 in San Francisco Square for the periods: (**a**) daytime 07:00–19:00; (**b**) evening 19:00–23:00; (**c**) night 23:00–07:00.

A query can also be established for the predetermined day–evening–night periods, as shown in Figure 16.

Appendix A contains different events that show examples of the activity of the city, namely: the garbage collection truck, the noise from construction work in a street, and the noise derived from a leisure activity—a bar.

## 4. Conclusions and Future Work

We have presented the design and implementation of a complete low-cost system, composed of nine sensors nodes, for a WASN deployed in the city of Linares (Jaén), Spain, which has been working continuously for ten months. The complete system has covered the network topology design, hardware and software of the sensor nodes, protocols, and a cloud web server platform. The information provided for the system for each location where the nodes have been deployed is as follows: L_Aeq_ for a given period of time; some noise indicators indicated in the END (L_den_, L_day_, L_evening_, and L_night_), the percentile noise levels (L_A01T_, L_A10T_, L_A50T_, L_A90T_, and L_A99T_); a temporal evolution representation of the noise levels; and the predominant frequency of the noise. Moreover, a map using the Google Maps platform API has been displayed, representing the L_Aeq_ in each location.

Before deploying the sensor nodes in the city, different experiments were conducted to verify the performance of the Arduino Due hardware, together with the software implemented for the acoustic noise monitoring. The results were compared to the measurements acquired using a commercial SLM, which proved that the sensor nodes have a very good performance. However, the dynamic range of the sensor nodes was 44–105 dBA, and therefore, they cannot measure noise levels below 44 dBA. This can distort the results for measurements in non-noisy environments and especially during the night measurement period, from 23:00–07:00.

The performance of the Arduino Due is very good; the sensor nodes have been running continuously for 10 months, monitoring noise every second and sending the parameters to the cloud web server every 30 s. Some sensor nodes have presented problems derived from power outages and the subsequent restart of the devices (i.e., they did not restart properly). To solve this issue, it was necessary to mobilize technicians for a manual restart. This is a clear inconvenience if frequent power outages occur. There have also been problems with sending the data to the private cloud web server when the corporate Wi-Fi network of the city council did not work. In addition, there have been multiple hacker attacks on the cloud web server, with attempts to make insertions to the MySQL database, which succeeded in some cases. Therefore, we must increase the level of security in the cloud server.

Alternately, the amount of data received and stored has been enormous, given that every 30 s, each node sends the measured parameters. This approach is fine for analyzing acoustic noise over a considerable period of time, but it is not feasible to maintain it sustainably for many months or years.

An analysis of the results obtained from the acoustic noise in the city indicates that the variability of the acoustic noise in a specific location is very low, and therefore, a continuous measurement for one month is more than enough to characterize the noise in that location.

Although much work remains in order obtain accurate maps of noise levels in smart cities using WASN, the proposed system presented in this work can serve as an excellent starting point for this task.

Future research should aim to improve the dynamic range of the sensor nodes to be able to monitor acoustic noise below 44 dBA, and design and incorporate a fog computing platform between the sensor nodes and the cloud so as to avoid data loss due to the lack of a connection to the cloud. Security should be included in the protocols for sending the data, for example, HTTP. In addition, because the noise perception is affected by subjective factors, and there is not a direct correlation between the indicators and the subjective perception of the noise, the implementation of a module that is capable of evaluating the subjective impact of the noise annoyance is also proposed as a future work.

## Figures and Tables

**Table 1:** Information provided by the system.

Parameter	Description
L_AeqT_	A-weighting equivalent continuous sound pressure level
L_den_	Day–evening–night level
L_day_	A-weighted average sound level over the daytime period 07:00–19:00
L_evening_	A-weighted average sound level over the evening period 19:00–23:00
L_night_	A-weighted average sound level over the night period 23:00–07:00
L_Amax_	Maximum A-weighted noise level during the measurement period
F_eqmax_	Predominant frequency (Hz) of the noise
L_A01T_, L_A10T_, L_A50T_, L_A90T_, and L_A99T_	Percentile noise levels, L_AnT_, which are defined as the A-weighted sound level that is exceeded n% of the measurement time interval

**Table 2:** The critical places to be monitored.

Id	Location	Id	Location
1	Andalucia Avenue	6	Cervantes Street
2	Ayuntamiento Square	7	Julio Burell Street
3	Isaac Peral Street	8	Ubeda Street
4	Santa Margarita Square	9	Noruega Street
5	San Francisco Square		

**Table 3:** The cost of the sensor nodes.

Description	Original Material Cost (€)	Compatible Material Cost (€)
Wi-Fi Sensor Node	45	23
Wi-Fi Sensor Node + Antenna MikroTiK SXT2	99	47
Sigfox Sensor Node	109	49
3G Sensor Node	90	48

**Table 4:** The sample length for a frequency-sampling rate of 33 kHz.

Frequency Sampling Rate	Type of Response	Time Capture	Sample Length
33 kHz	Fast	125 ms	4125
33 kHz	Slow	1 s	33,000

**Table 5:** The Arduino Due measurements in the lab.

Intensity	L_Aeq_ SLM (dBA)	Diff Arduino Due (dBA)
Intensity 1	60.5	0.13
Intensity 2	70.3	0.14
Intensity 3	86.8	0.16

**Table 6:** The SLM and Arduino Due measurements in an urban street.

Interval	L_Aeq_ SLM (dBA)	L_Aeq_ Arduino (dBA)	Diff (dBA)
Interval 1	60.59	60.71	0.12
Interval 2	60.01	60.90	0.89
Interval 3	60.85	61.44	0.59
Interval 4	61.15	61.70	0.55

**Table 7:** The SLM and Arduino Due measurements in an urban street for a full day.

Device	L_Aeq_ (dBA)	L_day_ (07:00–19:00)	L_evening_ (19:00–23:00)	L_night_ (23:00–07:00)
SLM	56.90	57.60	58.90	53.10
Arduino Due	57.96	57.83	61.31	53.83

**Figure A1:**
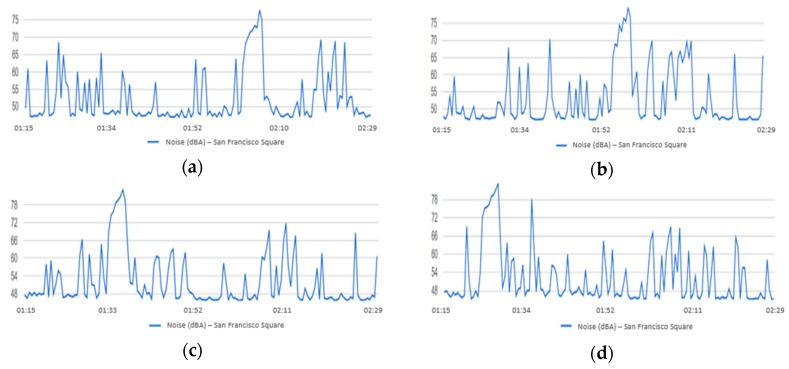
The temporal evolution of the noise in San Francisco Square for the period of 01:15–02:30 on the following days: (**a**) Monday 26 June 2017; (**b**) Tuesday 27 June 2017; (**c**) Wednesday 28 June 2017; (**d**) Thursday 29 June 2017.

**Figure A2:**
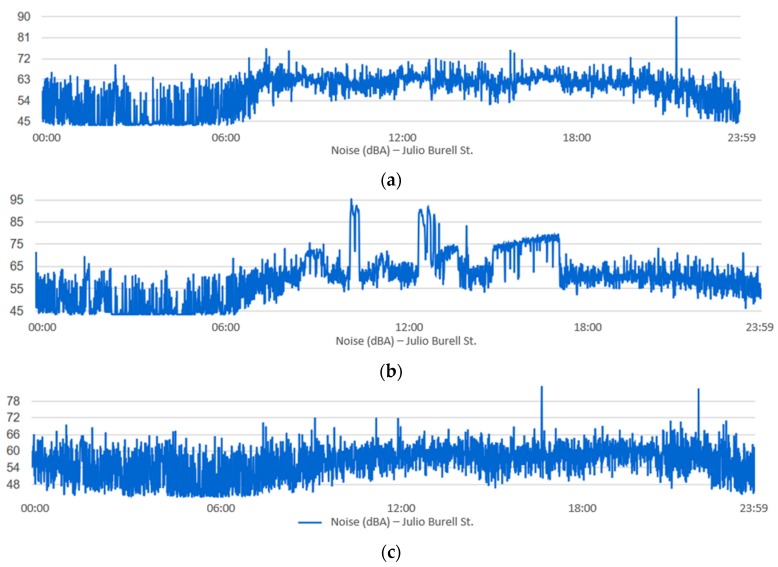

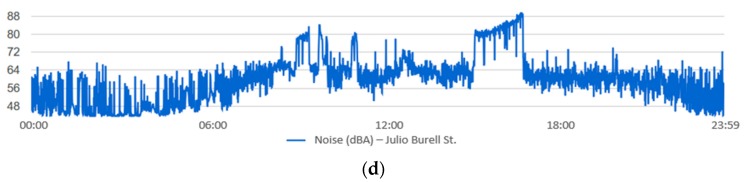
The temporal evolution of the noise at the Julio Burell St. location: (**a**) 15 November 2016, day without works under construction; (**b**) Friday 18 November 2016, starting the works under construction; (**c**) Sunday 20 November 2016, stopping the works (no labor on this day); (**d**) Monday 21 November 2016, restarting the works.

**Figure A3:**
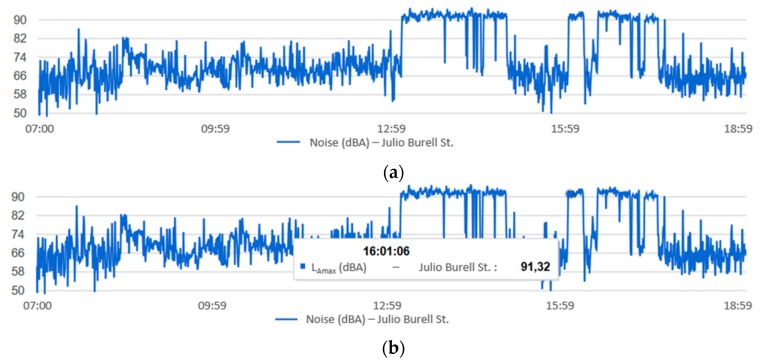
(**a**) The temporal evolution of the maximum noise at the Julio Burell St. location for the daytime period on 22 November 2016; (**b**) instant where the work resumes at 16:01.

**Figure A4:**
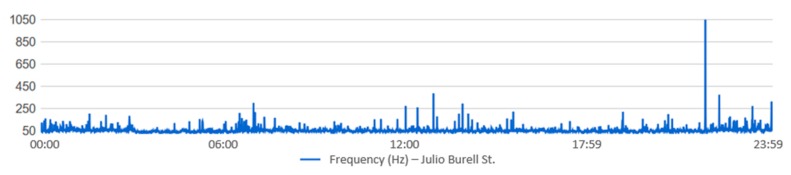
The predominant frequency during 15 November 2016, at Julio Burell Street.

**Figure A5:**
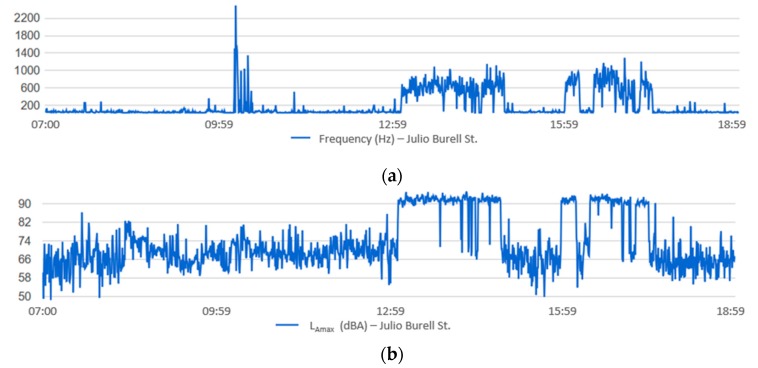
(**a**) The predominant frequency measured by the sensor node located at Julio Burell St. during 22 November 2016, in a daytime period. (**b**) Maximum noise measured in Julio Burell St. location for the daytime period on 22 November 2016.

**Figure A6:**
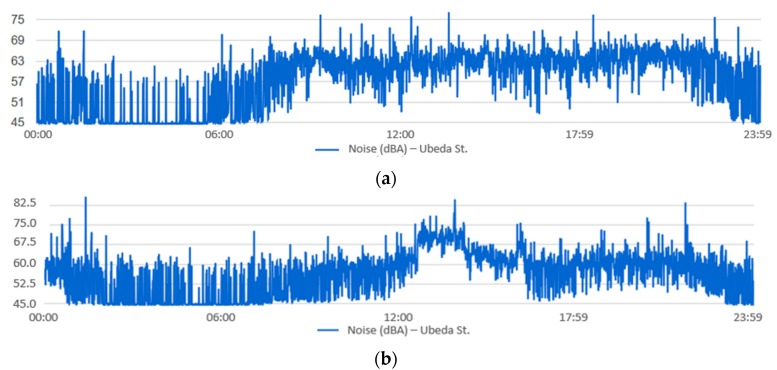
The temporal evolution of the noise in Ubeda Street during: (**a**) a weekday, 9 November 2016; (**b**) a weekend day, 13 November 2016.

**Figure A7:**
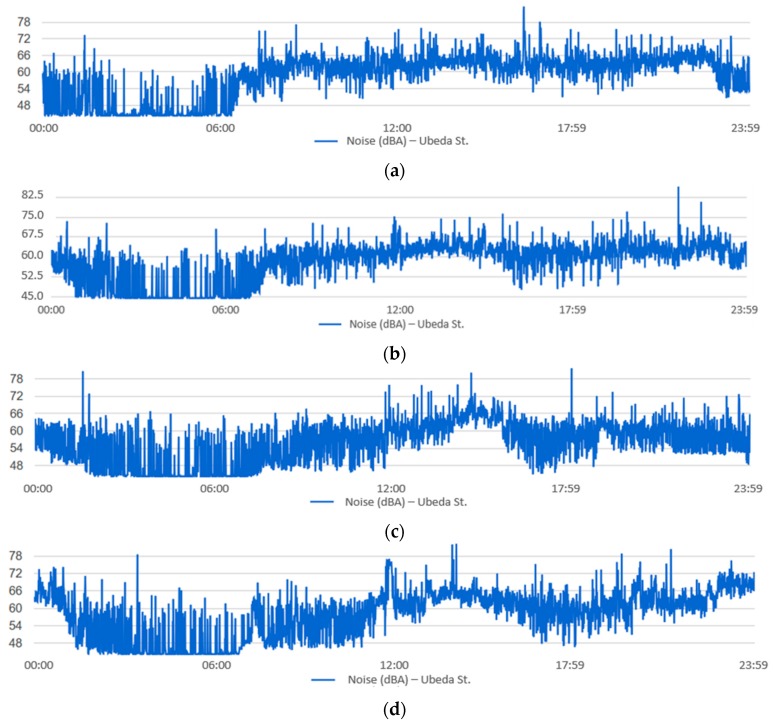
The temporal evolution of the noise in Ubeda Street during: (**a**) a weekday, 18 May 2017; (**b**) a weekday, 1 April 2016; (**c**) a weekend day, 3 May 2017; (**d**) a weekend day, 16 April 2017.

**Figure A8:**
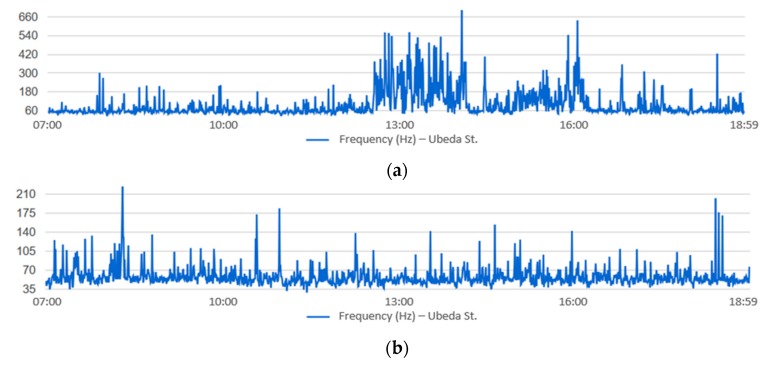
(**a**) The predominant frequency measured by the sensor node located in Ubeda Street on a weekend day, 13 November 2016, with the presence of people; (**b**) the predominant frequency measured by the sensor node on a weekday, 9 November 2016, without the presence of people.

## Appendix A

### Appendix A.1. Event 1—The Garbage Collection Truck

During the night period of the Figure 16, we appreciated an event of greater acoustic noise at approximately 01:30. As shown in Figure A1, if we visualize the period from 01:15–02:30 during some days of the week, we observed that the noise occurred periodically on all of the days, and lasted approximately five minutes. When we moved to the location to find the source of this noise, we could see that it was the garbage collection truck.

### Appendix A.2. Event 2—The Noise in the Works under Construction in a Street

Another event that we would like to highlight is how the sensor node, located in Julio Burell Street, detected the acoustic noise due to some works under construction, which were carried out for several days. Figure A2 shows the temporal evolution of noise on that street during two days, in the absence of works under construction and days where such works were carried out.

Figure A3 shows the maximum noise during 22 November 2016, for the daytime period (07:00–19:00) at Julio Burell Street. It can be seen that there is a permanent noise of approximately 92 dB from 13:00 to 15:00. At that time, the noise subsided, probably due to the break for the workers’ lunch, and the activity restarted at 16:00. We could say that the workers had an hour’s break and finished their workday at 17:30.

If we analyze the predominant frequency of noise during a full day without construction work, we can see that the frequency is lower than 100 Hz for most of the day, as shown in Figure A4.

However, if we now show the frequency on a day where construction work is being performed, we can see that when there is an increase in noise due to construction work, the predominant frequency increases, which could be due to some hammering. Figure A5 shows the predominant frequency against the maximum noise during 22 November 2016, during a daytime period (07:00–19:00) on Julio Burell Street.

### Appendix A.3. Event 3—The Noise Derived from a Leisure Activity (A Bar)

Finally, we show the measurements made by the sensor with Id eight located on Ubeda Street. As seen in Figure 10b, the sensor was located just above a terrace of a bar where people usually go to eat and drink outdoors in good weather. Figure A6 shows the temporal evolution of acoustic noise during a weekday (Wednesday, 9 November 2016) and during a weekend day (Sunday, 13 November 2016).

We can appreciate how the noise on a weekday began to increase at approximately 07:30, and remained at almost 63 dB until 21:00, at which point it began to decrease. However, on the weekend, it can be seen that the noise began to increase at approximately 12:45, and remained here until approximately 16:15, because it was a sunny day, and many people probably went to eat at that bar terrace.

This noise pattern is always repeated for weekdays and weekend days. Figure A7a,b shows the temporal evolution of several weekdays in different months (Thursday, 18 May 2017 and Tuesday, 11 April 2017), while Figure A7c,d shows the temporal evolution of the noise on two weekend days (Sunday 3 May 2017 and Sunday 16 April 2017). It should also be noted that during many Saturday nights, there was also noise from people, which can be inconvenient for neighbors, as shown in Figure A7d. The noise did not begin to decrease until 01:00 on Sunday 16 May 2017.

If the predominant frequency of the noise is shown, we can verify that the frequency is higher in that time slot because of the presence of people, as shown in Figure A8.
